# Enhancing both oral bioavailability and brain penetration of puerarin using borneol in combination with preparation technologies

**DOI:** 10.1080/10717544.2016.1259372

**Published:** 2017-02-06

**Authors:** Tao Yi, Dandan Tang, Fan Wang, Jiqiong Zhang, Jiao Zhang, Jirui Wang, Xiaoyu Xu, Jifen Zhang

**Affiliations:** 1 School of Health Sciences, Macao Polytechnic Institute, Macau, China,; 2 College of Pharmaceutical Sciences, Southwest University, Chongqing, China, and; 3 Chongqing Jiangjin Teacher Training School, Chongqing, China

**Keywords:** Puerarin, borneol, nanocrystals, inclusion compounds, self-microemulsifying drug delivery system

## Abstract

Now there are few good oral preparations of puerarin used in cerebrovascular diseases because of its poor oral absorption caused by the low water solubility and the poor penetration into brain. In this study, three oral formulations of puerarin, nanocrystals suspension (NCS), inclusion compounds solution (ICS) and self-microemulsifying drug delivery system (SMEDDS) were prepared with borneol as an oral brain-targeting enhancer. A rat syngeneic *in vitro* model of the brain–blood barrier (BBB) was established to investigate effects of borneol on the permeability of puerarin across the BBB. The pharmacokinetics of puerarin in mice after oral administration was investigated by a high performance liquid chromatography-mass spectrometry/mass spectrometry (HPLC-MS/MS) method. The *in vitro* BBB model study showed the permeability of puerarin was increased significantly (*p *< 0.05) and the value of transepithelial electrical resistance at 2 h was decreased significantly (*p *< 0.01) when the concentration of borneol was over 12.5 μg/mL compared with the control group. The pharmacokinetics results indicated borneol with doses of over 50 mg/kg could obviously increase both intestinal absorption and brain penetration of puerarin. With co-administration of borneol (100 mg/kg), the AUC of puerarin both in plasma (AUC_plasma_) and in brain (AUC_brain_) for SMEDDS were significantly higher than those for NCS (*p *< 0.01) and ICS (*p* < 0.05). These results suggested borneol in combination with SMEDDS could improve both the oral absorption and the brain penetration of puerarin in mice, which was promising for the development of an oral formulation of puerarin used in cerebrovascular diseases.

## Introduction

Puerarin is the main active ingredient in *Puerariae Radix*, a traditional Chinese medicine herb. Puerarin has many beneficial biological activities related to cerebrovascular conditions, such as improving microcirculation, increasing blood flow in the brain, neuroprotection, anti-thromboxane, anti-spasm and anti-platelet aggregation (Wei et al., 2014; Zhang et al., 2014). It has been widely used as a therapeutic agent to cerebrovascular diseases, especially those caused by cerebral ischemia (Wu et al., 2007; Tao et al., 2013). It could attenuate ischemia–reperfusion injury and promote function recovery of ischemic region by astrocyte apoptosis inhibition (Wang et al., 2014), cerebral vasodilation (Yan et al., 2009) and thrombolysis (Choo et al., 2002).

Clinical applications of puerarin were greatly restricted due to its adverse physicochemical properties. The ratio of AUC of cerebrospinal fluid and serum was only 2.92% after intravenous administration to rats (Wang, 2012) because of its poor penetration cross the brain–blood barrier (BBB) and being a substrate of P-glycoprotein (Cui et al., 2007), which may diminish its therapeutic effects on cerebrovascular diseases. Moreover, intravenous injection is the main clinical route of puerarin presently because of its poor oral absorption caused by low water solubility and poor intestinal permeability (Li et al., 2014). Additionally, the frequent intravenous administration of high doses due to the short elimination half-life of puerarin and a co-solvent of 1,2-propanediol in puerarin injection may lead to severe and acute side effects, such as allergy, hemolytic anemia and drug fever (Chen, 2010; Ma, 2010).

In order to deliver puerarin into the brain by oral administration, two obstacles should be overcome. One is the poor oral bioavailability and the other is the poor penetration into brain. For the first problem, many new formulations such as nanocrystals (Tu et al., 2013; Yi et al., 2015), inclusion compounds (Zhang et al., 2010; Liu et al., 2012) and microemulsions (Tang et al., 2013; Yu et al., 2013), have been used. However, their effects on oral absorption have not been compared. Whether these formulations could help puerarin cross the BBB was not clear, either. To resolve the second problem, borneol, a simple bicyclic monoterpene extracted from the resin and volatile oil of dipterocarp, has been used as an oral brain-targeting enhancer (Cai et al., 2014), which could improve oral bioavailability by accelerating drug absorption in gastrointestinal tract, i.e. salvianolic acids (Lai et al., 2011), gastrodin (Cai et al., 2014), and facilitate drug delivery into the brain by opening the BBB, i.e. geniposide (Chen et al., 2013; Yu et al., 2013), ganciclovir (Ren et al., 2013), kaempferol (Zhang et al., 2015). The mechanisms of opening the BBB by borneol may attribute to disassembly effects on tight junction integrity (Chen et al., 2013) and regulation of the expressions of mdr1a, mdr1b and mrp1 (Yu et al., 2013).

The aim of the present study was to develop an appropriate oral formulation of puerarin to enhance both oral bioavailability and brain penetration using borneol as an oral brain-targeting enhancer. The rat brain microvascular endothelial cells (BMECs) and astrocytes from the same genus were co-cultured to establish the *in vitro* BBB model. The effects of borneol on the transportion of puerarin across the BBB model were investigated. The dose of borneol was further selected by pharmacokinetic experiments *in vivo*. Three oral formulations of puerarin, nanocrystals suspension (NCS), inclusion compounds solution (ICS) and self-microemulsifying drug delivery system (SMEDDS) were prepared with borneol and the pharmacokinetic behaviors of puerarin in mice blood and brain after oral administration were compared.

## Materials and methods

### Materials

Borneol (purity > 98%) was purchased from Chongqing Peace Chain-Drugstore Co., Ltd. (Chongqing, China). Puerarin (purity > 98%) was purchased from Sichuan Yuxin Pharaceutical Co., Ltd. (Chengdu, China) and parahydroxy benzaldehyde (internal standard, IS, purity > 98%) was purchased from the National Institute for the Control of Pharmaceutical and Biological Products (Beijing, China). Capmul MCM was kindly given by Abitec Co. (Janesville, WI). Labrasol was donated by Gattefosse (FRA). Polysorbate 80 (Tween 80) was purchased from Chengdu Kelong Chemical reagent Co., Ltd. (Chengdu, China). Hydroxypropyl-β-cyclodextrin (HP-β-CD, molecular weight of 1447, degree of substitution 5.4%) was purchased from Hubei Haiboyuan Chemical Co., Ltd. (Wuhan, China). HPLC-grade methanol was purchased from Fisher Chemicals Co. (Pittsburgh, PA). HPLC grade formic acid was purchased from Tedia Company Inc. (Fairfield, OH). DMEM-F12 medium was purchased from GIBCO (Invitrogen Corporation, Waltham, MA). Trypsin (1:250) and L-glutamine were purchased from Amresco (USA). Fetal bovine serum was purchased from Hyclone (Thermo Scientific, USA). EDTA and gelatin were purchased from Glenview (Glenview Scientific Inc., USA). Collagenase type II, bovine serum albumin, poly-L-lysine (molecular weight of 70 000–150 000) were purchased from Sigma-Aldrich Co. LLC. (USA). All the antibodies used in this study were from Santa Cruz (USA) except anti-GAPDH monoclonal antibody which was from ZSGB-BIO (Beijing, China). All the kits in this study were purchased from ZSGB-BIO (Beijing, China). Sodium fluorescein (SF) was purchased from Sigma (Sigma-Aldrich Co. LLC., USA). Other solvents and chemicals were of analytical grade.

### Animals

Kuming mice (weight of 25–30 g, half male and half female) of clean grade were purchased from Chongqing Academy of Chinese Materia Medica. Newborn Sprague-Dawley (SD) rats of indicated days were obtained from the Experimental Animal Center, Chongqing Medical University (Chongqing, China). All animals were housed in the Experimental Animal Center, College of Pharmaceutical Sciences & College of Chinese Medicine, Southwest University (Chongqing, China). All the experiments were performed in accordance with China’s Guidelines for Care and Use of Laboratory Animals which was approved in 1998.

### Effects of borneol on the transport of puerarin across the *in vitro* BBB model

#### Cytotoxicity assays

The *in vitro* BBB model was established by co-culturing primary rat BMECs and cerebral astrocytes on opposite side of Transwell membrane inserts as described in previous reports (Xue et al., 2013). The integrity of the *in vitro* BBB model was checked by transepithelial electrical resistance (TEER) and permeability studies of SF. The method and evaluation *of BBB* were listed in Appendix A in Supplementary data.

In order to find an optimal range of borneol concentrations on the *in vitro* BBB model, the cytotoxicity of puerarin and borneol on primary rat BMECs and cerebral astrocytes was evaluated by the methyl thiazolyl tetrazolium (MTT) colorimetric assay. The cells with a confluence of 80–90% were seeded at a density of 5 × 10^5^ cells per well in 96-well plates. After culture for 24 h, the culture medium was removed and 1 mL of D-Hanks containing 100 μg/mL puerarin and different concentrations of borneol in the range of 1–200 μg/mL were added into the wells. An aliquot of D-Hanks was added for negative control wells. The cells were further cultured for 4 h. Subsequently, 20 μL of 5 mg/mL MTT PBS solution were added into each well, and then the cells were stained at 37 °C for 4 h. Thereafter, the medium was removed, and the cells were mixed with 150 μL of dimethyl sulfoxide (DMSO). The absorbance was measured at 490 nm with a Multiskan Go Microplate reader (Thermo, USA). Relative cell viability (*R*%) was calculated as follows: *R*% = absorbance_test_/absorbance_control _× 100%

#### Effects of borneol on the transport of puerarin across the in vitro BBB model

After 7 days of co-culturing of primary rat BMECs and cerebral astrocytes, the *in vitro* BBB model was established. The permeability of puerarin across the *in vitro* BBB model with the action of borneol was evaluated. The experiments were conducted at 37 °C by adding 1 mL of HBSS with 100 μg/mL puerarin and different concentrations of borneol in the range of 1–200 μg/mL on the apical side and 2 mL of HBSS on the basolateral side. After 15, 30, 60, 90 and 120 min of incubation, samples were collected from the basolateral side and puerarin concentration was measured by HPLC. *P*_app_ was calculated as follows: *P*_app _=_ _d*Q/*d*t* × 1/*AC*_0._

Where d*Q/*d*t* is the transport rate (μg/s), *C*_0_ is the initial drug concentration on the apical side (μg/mL), and *A* is the surface area of the membrane filter (cm^2^). At 120 min, TEER of the BBB model was also measured.

### Preparation and characterization of different formulations

#### Borneol solution

To get a borneol solution with concentration of 10, 20 or 40 mg/mL, borneol of 1, 2 or 4 g respectively was added into 100 mL of 50% (v/v) alcohol and sonicated until borneol was dissolved completely.

#### NCS of puerarin

The NCS of puerarin was prepared by an ultrasonic method. The concentration of puerarin, the ultrasonic magnitude and time were optimized by the particle size of nanocrystals and the stability of suspension. The optimum preparation method was as follows: puerarin of 1 g was dispersed into 100 mL pure water under stirring and then was ultrasonicated with an Ultrasonic cell crusher equipped with a 10 mm shaft working (Ningbo Xinzhi Biotechnology Co., Ltd., China) at 800 W for 3 min with 5 s sonication and 5 s standby. An NCS was achieved, whose particle size was 127.3 ± 3.53 nm and zeta potential was −27.5 ± 2.42 mV (Zetasizer Nano-ZS, Malvern Instruments, Malvern, UK).

#### SMEDDS of puerarin

The formulation of SMEDDS was screened by test of solubility, compatibility of oil and surfactant, and pseudo-ternary phase diagram, and then was optimized by particle size, self-microemulsifying time and drug loading. Finally, the SMEDDS of puerarin was prepared as follows: Capmul MCM of 10 g, Tween 80 of 16 g, Labrasol of 4 g and 1,2-propylene glycol of 20 g were mixed and stirred on a magnetic stirrer for 20 min at 37 °C. Then puerarin of 0.5 g and borneol of 0.25 g were added into the resultant mixture and continuously stirred until puerarin and borneol were both dissolved completely.

The self-microemulsifying time was less than 30 s. The particle size of resultant microemulsions was151.6 ± 1.92 nm and the zeta potential was −4.73 ± 0.38 mV (Zetasizer Nano-ZS, Malvern Instruments, Malvern, UK). The contents of puerarin and borneol were 10 mg/mL and 5 mg/mL respectively. The appearance, content of drugs, particle size, and self-microemulsifying time had no obvious changes under a storage of 6 months at room temperature.

#### ICS of puerarin

According to our previous study (Tang et al., 2014), the inclusion compounds were prepared as follows: HP-β-CD of 40 g was dissolved in 100 mL pure water, after which, puerarin of 0.5 g and borneol of 0.25 g were added and ultrasonicated for 30 min to get a clear solution. Both puerarin and borneol were incorporated into the cavity of HP-β-CD completely (Tang et al., 2014). The contents of puerarin and borneol were 10 mg/mL and 5 mg/mL, respectively.

### Pharmacokinetic studies in mice

Randomized design was applied to divide 324 mice into six groups. Prior to the experiment, the mice were kept fasten overnight. For each group, different formulations were administered by gavage as follows:

- 50% (v/v) alcohol solution (2.5 mL/kg) followed by puerarin NCS immediately (200 mg/kg), which was used as the control group.
- 10 mg/mL borneol solution (25 mg/kg) followed by puerarin NCS immediately (200 mg/kg);
- 20 mg/mL borneol solution (50 mg/kg) followed by puerarin NCS immediately (200 mg/kg);
- 40 mg/mL borneol solution (100 mg/kg) followed by puerarin NCS immediately (200 mg/kg);
- SEMDDS co-loading borneol (100 mg/kg) and puerarin (200 mg/kg);
- ICS co-loading borneol (100 mg/kg) and puerarin (200 mg/kg).

The Groups A, B, C and D were designed to validate the effects of borneol on oral absorption and the BBB penetration of puerarin. The Groups D, E and F were designed to compare the influences of preparation technologies on oral absorption and the BBB penetration of puerarin. The dose of 200 mg/kg for puerarin was selected according to the previous reports (Tang et al., 2013; Tu et al., 2013).

For each group, 6 mice were picked up at 10, 30, 45 min, and 1, 2, 4, 6, 8, 12 h after oral administration, respectively. The blood samples were collected into heparinized tubes. The animals were then sacrificed and brain tissues were separated. The blood sample was centrifugated at 3000 rpm for 15 min and the plasma was kept immediately at −80 °C until analysis. Each collected brain tissue was homogenized (F6/10-6G homogenizer, FLUKO (Shanghai) fluid machinery manufacturing Co., Ltd., Shanghai, China) with normal saline of 2-fold volume at 25 000 rpm for 1 min. The obtained brain homogenates were then centrifugated at 10 000 rpm for 10 min and the supernatant was kept immediately at −80 °C until analysis.

### HPLC-MS/MS analysis

For analysis, 5 μL IS solution (7600 ng/mL), 175 μL methanol and 375 μL acetonitrile were added to 100 μL plasma or brain homogenate sample and vortex-mixed for 2 min. The mixtures were centrifuged at 15 000 rpm for 10 min. The supernatants were dried vacuumly at 40 °C. The residues left were reconstituted in 100 μL methanol and centrifuged at 15 000 rpm for 10 min. The supernatant was analyzed using HPLC-MS/MS method.

Analysis of each sample was performed on a LC-20A HPLC system (Shimadzu Corporation, Japan) equipped with a Agilent Eclipse plus C18 column (100 mm × 4.6 mm id, 3.5 μm particle size, Agilent Technologies, USA) eluted with methanol and 10 mmol/L ammonium acetate containing 0.1% (v/v) formic acid (80:20, v/v) at a flow rate of 0.6 mL/min. The injection volume was 5 μL and the column temperature was maintained at 30 °C. The total chromatographic run time was 4.0 min.

The mass spectrometry system was an API4000 spectrometer (AB Corporation, USA) operating in the negative ionization mode. Quantitations were performed using the multiple reaction monitoring (MRM) mode to monitor the protonated precursor → production transition at m/z 415.0 → 295.0 for puerarin and at m/z 120.9 → 91.8 for the IS, respectively.

Method validation was done by evaluating a series of method-performance characteristics, such as selectivity, linearity, LOQ, accuracy, precision, matrix effect and stability according to the criteria suggested by the USFDA (the U.S. Food and Drug Administration). The other optimized parameters of the mass spectrometry and results of method validation were listed in Appendix B in Supplementary data.

### Pharmacokinetic data analysis

The data were analyzed according to the previous papers (Li et al., 2016; Rezazadeh et al., 2016). For each group, the concentration-time curve was profiled according to the mean concentration of 6 mice for a point. When calculating the pharmacokinetic parameters, one concentration at each time point was used to form a set of concentration-time data. Pharmacokinetic parameters, including the maximum concentration (*C*_max_), the time to reach *C*_max_ (*T*_max_), the area under the drug concentration versus time curve from zero to 12 h (AUC_0–12 h_), *t*_1/2_ in both plasma and brain were calculated using noncompartmental model by Drug Analysis System 2.0 (DAS 2.0) software package (Mathematical Pharmacology Professional Committee of China, Shanghai, China). The ratio of AUC_brain_ to AUC_blood_ was calculated.

All data were expressed as mean ± SD. One-way ANOVA was used to test the differences between groups and *p* < 0.05 or *p* < 0.01 was considered as significant difference.

## Results and discussion

### Effects of borneol on the transport of puerarin across the *in vitro* BBB model

The relative cell viability was all more than 90% when the concentration of puerarin was 100 μg/mL and the concentration of borneol was between 1 and 200 μg/mL, which implied no significant toxicity toward the BBB model. The transport results of puerarin across the BBB model with different amounts of borneol were shown in Table 1. The *P*_app_ of puerarin in the presence of 6.25 μg/mL of borneol was not significantly different from the control group without borneol. When the concentration of borneol was over 12.5 μg/mL, the *P*_app_ of puerarin increased significantly (*p* < 0.01). The change tendency for TEER of the BBB model at 120 min was in accord with that for *P*_app_ of puerarin: when the concentration of borneol was 6.25 μg/mL, the TEER was similar with the control group; when borneol was over 12.5 μg/mL, the TEER significantly decreased by more than 50% (*p *< 0.01). The decrease of TEER meant that the tight junction of the BBB was loosed by borneol, consequently prompting the transport of puerarin across the BBB. However, when the concentration of borneol was increased from 12.5 to 100 μg/mL, there were obvious differences neither for the *P*_app_ of puerarin nor for the TEER of the BBB model.

**Table 1.** The *P*_app_ of puerarin and the TEER of the BBB model at 120 min associated with the action of borneol (*n *= 3).

Concentration of borneol (μg/mL)	0 (the control group)	6.25	12.5	25	50	100
*P*_app_ (×10^−6^ cm·s)	1.9 ± 0.5	1.7 ± 0.8	13.2 ± 0.2^a^	13.1 ± 0.1^a^	13.8 ± 1.9^a^	11.1 ± 1.8^a^
TEER (Ω·cm^2^)	221.0 ± 21.1	204.0 ± 21.5	99.1 ± 9.1^a^	102.4 ± 7.2^a^	93.6 ± 7.5^a^	95.7 ± 7.2^a^

^a^*p* < 0.01 compared with the control group not containing borneol.

### Effects of borneol on the oral absorption and the brain penetration of puerarin

**Figure 1.**
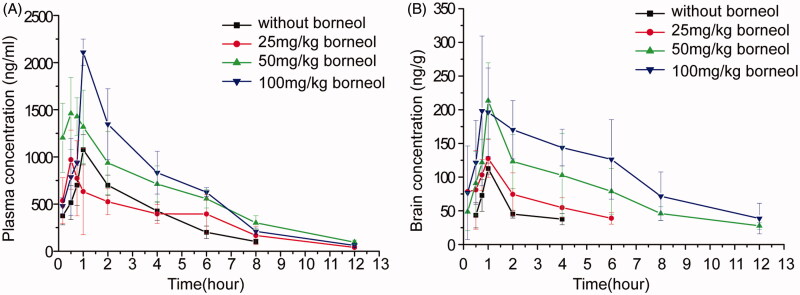
Mean concentration-time curves of puerarin in (A) plasma and (B) brain after administration of puerarin (200 mg/kg) NCS without or with different dose of borneol (25, 50, 100 mg/kg) in mice (*n* = 6).

To investigate the effects of borneol dosage on oral absorption and brain penetration of puerarin, NCS of puerarin without any stabilizers was used under the following considerations: (a) the puerarin concentration in brain was too low to be detected after oral administration of the coarse powder suspension of puerarin; and (b) stabilizers added may influence the oral absorption and brain penetration of puerarin. The concentration profiles of puerarin in plasma and brain after administration of puerarin (200 mg/kg) NCS without or with different dose of borneol (25, 50, 100 mg/kg) were presented in Figure 1. The major pharmacokinetic parameters of puerarin in plasma and brain calculated by non-compartment model were presented in Tables 2 and 3, respectively.

**Table 2.** Pharmacokinetic parameters of puerarin in mice plasma after administration of puerarin (200 mg/kg) NCS without or with different dose of borneol (25, 50, 100 mg/kg) (*n* = 6).

Parameters	Puerarin NCS without borneol	Puerarin NCS with borneol of 25 mg/kg	Puerarin NCS with borneol of 50 mg/kg	Puerarin NCS with borneol of 100 mg/kg
AUC_(0 − 12 h)_ (ng/mL*h)	3511.42 ± 583.86	3877.15 ± 367.46	6788.15 ± 1288.18^b^^,^^d^	7594.67 ± 649.29^b^^,^^d^
*t*_1/2_ (h)	1.90 ± 0.32	2.55 ± 0.65	2.76 ± 0.27^b^	1.93 ± 0.41^e^
*T*_max_ (h)	1.00 ± 0.00	0.69 ± 0.24^a^	0.60 ± 0.14^b^	1.00 ± 0.00^d^^,^^f^
*C*_max_ (ng/mL)	1076.02 ± 160.52	1264.67 ± 121.95	1645.25 ± 193.03^b^^,^^c^	2108.22 ± 140.54^b^^,^^e^

^a^*p* < 0.05 compared with the control group without borneol.

^b^ *p* < 0.01 compared with the control group without borneol.

^c^ *p* < 0.05 compared with the group of 25 mg/kg of borneol.

^d^ *p* < 0.01 compared with the group of 25 mg/kg of borneol.

^e^ *p* < 0.05 compared with the group of 50 mg/kg of borneol.

^f^ *p* < 0.01 compared with the group of 50 mg/kg of borneol.

**Table 3.** Pharmacokinetic parameters of puerarin in mice brain after administration of puerarin (200 mg/kg) NCS without or with different dose of borneol (25, 50, 100 mg/kg) (*n* = 6).

Parameters	Puerarin NCS without borneol	Puerarin NCS with borneol of 25 mg/kg	Puerarin NCS with borneol of 50 mg/kg	Puerarin NCS with borneol of 100 mg/kg
AUC_0–12 h_ (ng/mL × h)	214.13 ± 22.05	391.76 ± 80.91^a^	941.46 ± 310.30^a^^,^^c^	1303.75 ± 387.47^b^^,^^c^
*t*_1/2_ (h)	2.57 ± 0.69	2.72 ± 0.52	4.03 ± 0.76	4.30 ± 1.63
*T*_max_ (h)	1 ± 0	0.83 ± 0.29	1.00 ± 0	1.13 ± 0.60
*C*_max_ (ng/mL)	112.66 ± 9.94	143.93 ± 12.58	213.34 ± 47.57^a^	220.25 ± 69.29^a^

^a^*p* < 0.05 compared with the control group without borneol.

^b^ *p* < 0.01 compared with the control group without borneol.

^c^ *p* < 0.05 compared with the group of 25 mg/kg of borneol.

Figure 1(A) displayed that the oral absorption of puerarin could be enhanced by borneol. Table 3 summarized that 1.93 and 2.16-fold increase in AUC_blood_ (AUC_0–12 h_ in blood) of co-administration with borneol (50 mg/kg and 100 mg/kg) were observed, respectively (*p *<* *0.01). The mean values of *C*_max_ for co-administration of borneol of 50 mg/kg and 100 mg/kg were 1.53 and 1.96 times, respectively, greater than that of the control group without borneol (*p *<* *0.01). However, there were no obvious differences for AUC_blood_ or *C*_max_ between the control group and the group of co-administration of borneol (25 mg/kg). The results suggested that the enhancing effect of borneol on the oral absorption of puerarin was dose-dependent, which was in accord with a previous study (Xiao et al., 2007). After oral administration of 15, 30 and 90 mg/kg of borneol, the oral bioavailability of tetramethylpyrazine phosphate in plasma was 1.52, 2.21 and 2.95 times increase, respectively (Xiao et al., 2007).

Table 3 illustrated that compared with the control group, the AUC_brain_ (AUC_0–12 h_ in brain) for co-administration of borneol was improved by 0.83-, 3.39- and 5.08-fold for 25, 50 and 100 mg/kg of borneol, respectively. The *C*_max_ of puerarin was increased by 33.08%, 89.36% and 95.50% for 25, 50 and 100 mg/kg of borneol, respectively. Figure 1(B) showed the puerarin in brain could not be detected after 4 h of administration of puerarin NCS alone, whereas it could be detected even after 12 h with co-administration of borneol (50 mg/kg and 100 mg/kg). The ratio of AUC_brain_ to AUC_blood_ was 1.66, 2.27 and 2.81 for co-administration of borneol at the dose of 25, 50 and 100 mg/kg, respectively. These results suggested that borneol could prompt the transportation of puerarin across the BBB in a dose-dependent manner. Similar dose–effect relationship was also observed by Dong et al. (2012). Rats were intragastrically administrated with 50, 100, 200, and 400 mg/kg of borneol, and then injected with geniposide. The increasing effect of borneol on the penetration of geniposide across the BBB was most obvious when the dose of borneol was 200 mg/kg.

The mechanisms of borneol increasing the oral absorption and brain distribution of puerarin were not fully understood. Borneol could alter the lipid phase of the intestinal mucous membrane, accelerate the fluidity of the polar head group regions of cell membranes and loosen the intercellular tight junction in intestinal tract. Our cell experiment *in vitro* demonstrated that borneol could reduce intercellular tight junction of the BBB. The disassembly effect on tight junction integrity should be an important mechanism. Another possible mechanism may be attributed to P-glycoprotein inhibition of borneol. Borneol could inhibit P-glycoprotein significantly in MDCK, Hela cells and the intestine cell (Chen & Wang, 2003; He et al., 2011). As a P-glycoprotein substrate (Luo et al., 2011), the P-glycoprotein-mediated efflux of puerarin was likely to be reduced by borneol in the intestinal tract and brain.

### Effects of different formulations on oral absorption and brain penetration of puerarin

**Figure 2.**
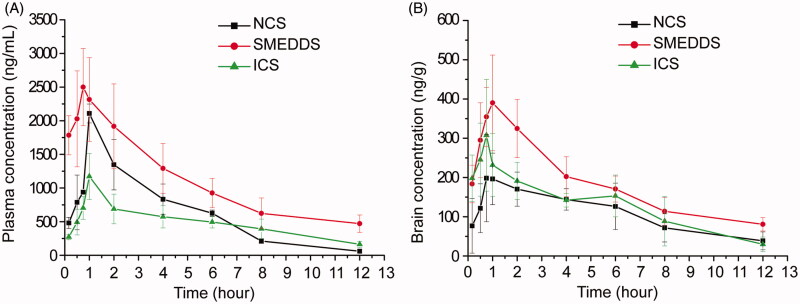
Mean concentration-time curves of puerarin in (A) plasma and (B) brain after administration of NCS, ICS and SMEDDS containing both puerarin (200 mg/kg) and borneol (100 mg/kg) in mice (*n* = 6).

The concentration profiles of puerarin in plasma and brain after oral administration of NCS, ICS and SMEDDS containing both puerarin (200 mg/kg) and borneol (100 mg/kg) were presented in Figure 2. The major pharmacokinetic parameters of puerarin were presented in Tables 4 and 5.

**Table 4.** Pharmacokinetic parameters of puerarin in mice plasma after administration of NCS, ICS and SMEDDS containing both puerarin (200 mg/kg) and borneol (100 mg/kg) (*n* = 6).

Parameters	NCS	SMEDDS	ICS
AUC_(0–12 h)_ (ng/mL × h)	7594.67 ± 649.29	13 174.16 ± 2487.95^b^	5783.78 ± 1253.09^c^
*t*_1/2_ (h)	1.93 ± 0.41	4.34 ± 1.28^a^	4.69 ± 0.45^b^
*T*_max_ (h)	1.00 ± 0.00	0.83 ± 0.20	0.96 ± 0.10
*C*_max_ (ng/mL)	2108.22 ± 140.54	2844.30 ± 395.32^a^	1217.02 ± 288.03^b^^,^^c^

^a^*p* < 0.05 compared with NCS.

^b^ *p* < 0.01 compared with NCS.

^c^ *p* < 0.01 compared with SMEDDS.

**Table 5.** Pharmacokinetic parameters of puerarin in mice brain after administration of NCS, ICS and SMEDDS containing both of puerarin (200 mg/kg) and borneol (100 mg/kg) (*n* = 6).

Parameters	NCS	SMEDDS	ICS
AUC_(0–12 h)_ (ng/mL × h)	1303.75 ± 387.47	2199.81 ± 328.98^b^	1488.09 ± 299.92^c^
*t*_1/2_ (h)	4.30 ± 1.63	5.71 ± 1.03	2.20 ± 0.49^a^^,^^d^
*T*_max_ (h)	1.13 ± 0.60	0.85 ± 0.22	0.75 ± 0.18
*C*_max_ (ng/mL)	220.25 ± 69.29	462.48 ± 55.75^b^	385.94 ± 84.61^a^

^a^*p* < 0.05 compared with NCS.

^b^ *p* < 0.01 compared with NCS.

^c^ *p* < 0.05 compared with SMEDDS.

^d^ *p* < 0.01 compared with SMEDDS.

Figure 2 clearly illustrated the differences among the three formulations. Table 4 demonstrated that the AUC_blood_ for SMEDDS was significantly higher than those for NCS (*p* < 0.01) and ICS (*p* < 0.01), whereas there was no difference between NCS and ICS. The *C*_max_ of blood for SEMDDS was 0.35 and 1.34 times higher than those for NCS (*p* < 0.05) and ICS (*p* < 0.01), respectively. The *t*_1/2_ for SMEDDS and ICS were 4.34 h and 4.69 h, which were 2.25 and 2.43 times that for NCS, respectively. These results above verified that the ability of SEMDDS to enhance the oral absorption of puerarin and delay its elimination was much better than those of NCS and ICS. This may be attributed to the following reasons: (a) the intestinal lymphatic transport was a major contributor to the high oral bioavailability of microemulsions (Wu et al., 2011; Tang et al., 2013); (b) the large amount of surfactant could insert into the cell membrane and destroy the structure of lipid bilayers, thus altering membrane fluidity or permeability of the gastrointestinal tract; surfactant also could block members of the multidrug resistance protein-associated family, including P-gp (Li et al., 2011; You et al., 2014); and (c) the small size of emulsion droplets could enlarge the distribution of drugs in intestine.

The ratio of AUC_brain_ to AUC_blood_ was 0.148, 0.173 and 0.242 for NCS, SMEDDS and ICS, respectively. This meant that ICS was more effective in prompting puerarin to cross the BBB into brain than NCS and SMEDDS. A few studies have showed that cyclodextrins could be used as an enabling excipient for brain targeting (Brewster et al., 2004; Zirar et al., 2008; Jeulin et al., 2009). The amount of ribavirin in the brain was significantly higher when the drug was complex with alpha-cyclodextrin (Jeulin et al., 2009). The hydroxypropyl-β-cyclodextrin inclusion complex could concentrate more melarsoprol into the brain than nanosuspensions (Zirar et al., 2008). Cyclodextrins could extract the cholesterol from brain capillary endothelial cells leading to a reduction of the P-gp activity (Tilloy et al., 2006), which could increase the delivery of P-gp substrates, such as puerarin. Cyclodextrins could also decrease the expression and localization of tight junction protein occludin and damage the barrier integrity of brain endothelial cells, which could increase the permeability of the BBB (Vecsernyés et al., 2014).

However, after administration the same dose of puerarin and borneol, the AUC_brain_ for ICS was not the highest among the three formulations. The AUC_brain_ of SMEDDS was significantly higher than that of NCS (*p *< 0.01) and ICS (*p *< 0.05). The *C*_max_ in brain for SMEDDS was also the highest among the three formulations. *t*_1/2_ of SMEDDS was increased by 159.54% compared with ICS (*p *< 0.01) and was not obviously different from NCS. Considering that enhanced AUC and *C*_max_ as well as prolonged *t*_1/2_ in brain were beneficial to the therapy of cerebrovascular diseases, SEMDDS should be the preferable formulation for the oral administration of puerarin for cerebrovascular diseases.

## Conclusions

In the present study, the effects of borneol and three formulations (SMEDDS, NCS and ICS) on the oral absorption and penetration across the BBB of puerarin were investigated. Borneol could not only improve the oral bioavailability of puerarin but also increase the permeability of the BBB, thus enhancing the distribution of puerarin in brain tissue dose-dependently. SMEDDS co-loading borneol and puerarin gave the highest AUC_brain_ of all formula, which was 10.27 times that of puerarin NCS without borneol. So borneol in combination with self-microemulsifying technology was effective and promising for the oral delivery of puerarin to brain for brain diseases.

## Supplementary Material

doc_ZipFileToDownload_Appendix_B__Supplementary_data_for_HPLC_MSMS_analysis_docx.docx

Appendix_A._Supplementary_method_for_the_BBB.doc

## Notes

**Supplementary materials available online**
