# Supplementary material for: Enhancing both oral bioavailability and brain penetration of puerarin using borneol in combination with preparation technologies
Source: Drug Deliv. 2017 Feb 6;24(1):422–9. doi: 10.1080/10717544.2016.1259372 (PMC8241152; doi:10.1080/10717544.2016.1259372)
Supplement: doc_ZipFileToDownload_Appendix_B__Supplementary_data_for_HPLC_MSMS_analysis_docx.docx [file IDRD_A_1259372_SM7166.docx]

***Appendix B. Supplementary data for HPLC–MS/MS analysis***

Puerarin in plasma and brain samples was detected by HPLC–MS/MS method. The conditions of Mass spectrometry was shown in Table 1.

Table 1 The conditions of Mass spectrometry

| Determined substance | DP | CE | Entrance potential | Collision cell exit potential | Collision gas | Curtain gas | Ion spray voltage | Temperature |
| --- | --- | --- | --- | --- | --- | --- | --- | --- |
| Puerarin | -85 | -31 | -10.0 | -15 | 6 | 15 | -4500 | 550 |
| IS | -65 | -30 | -10.0 | -15 | 6 | 15 | -4500 | 550 |

The full-scan mass spectrums of puerarin and IS were shown in Figure 1. The typical chromatograms of blank samples, standard solutions and real samples were shown in Figure. 2. The retention times of puerarin and IS were 1.79 and 2.09 min respectively. There was no interference in the determination of the compounds in the analyses. The results suggested that a good selectivity was developed both for plasma and brain homogenate.


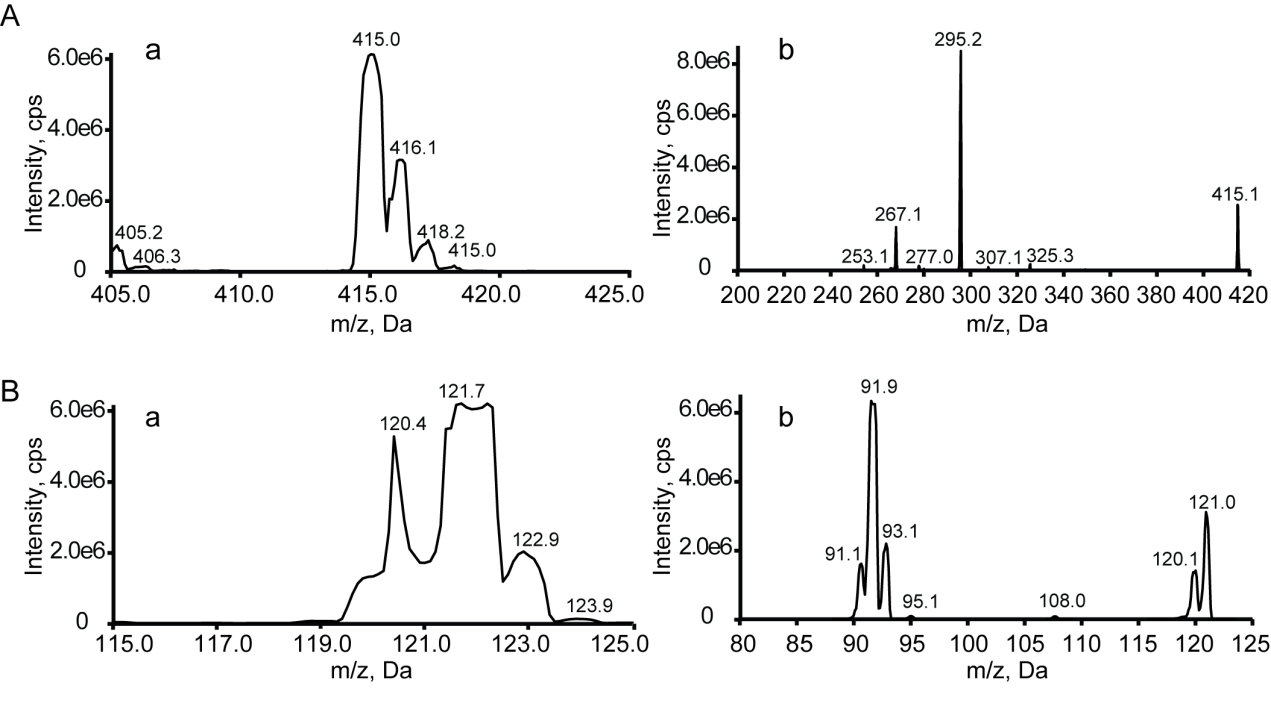
Figure 1. Full-scan mass spectra of [M+NH4]+ (A) and production spectra (B) of puerarin (a) and the IS (b).


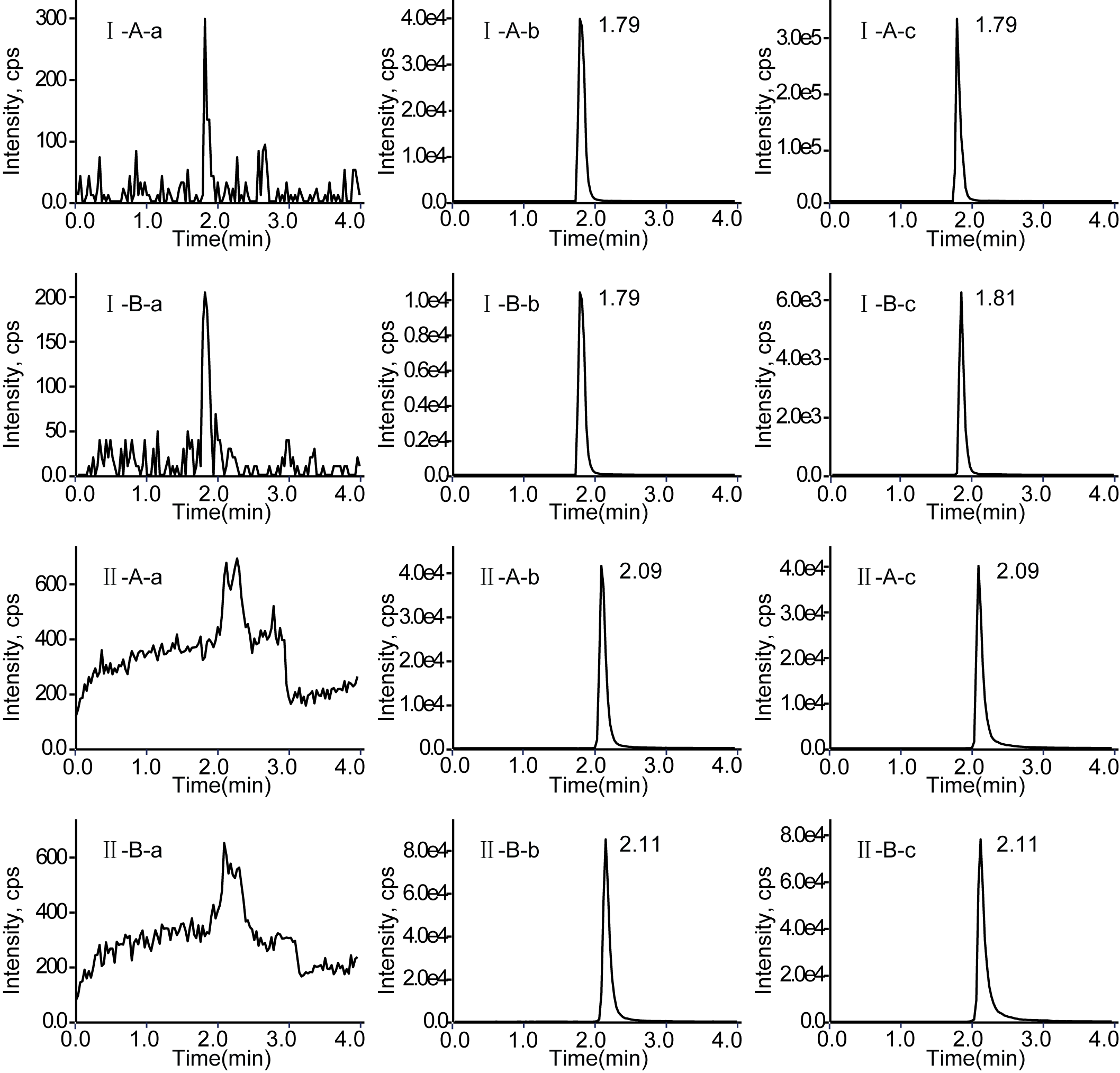
Figure 2. Representative MRM chromatographs of puerarin (I) and the IS (II) in plasma (A) and brain homogenate (B): (a) blank sample, (b) 100μL blank sample spiked with 5 μL puerarin (2252 ng/mL) and 5 μL IS (7600 ng/mL), (c)sample collected at 30 min after oral administration of puerarin nanocrystals suspension (200 mg/kg).

The calibration curves of puerarin in plasma and brain homogenate displayed good linear relationships in the concentration range of 56.3–11260 ng/mL (*r*≥0.999) and 28.15–2252 ng/mL (R≥0.999) respectively. The LOQ of puerarin in plasma and brain homogenate were 56.3 ng/mL and 28.15 ng/mL respectively. The intra- and inter-day accuracies and precisions were all within ±15%, as shown in Table 2. Both precision and accuracy were acceptable for bioanalysis. The CV of IS-normalized matrix factor were 9.2% and 12.7% for plasma and brain homogenate respectively at LLOQ, which were both below 15%. The stability of QC samples was assayed in three conditions, i.e. freeze-thaw for three cycles, room temperature for 24 h and −80℃ for 30 days. The remaining percentage ranged between 90.2% and 103.7% and the results suggested that puerarin was stable throughout the stability study. The developed method was convenient, sensitive with good linearity. The short analysis time of 4 min made this method highly efficient, suitable for batch analysis of numerous samples.

Table 2 The intra- and inter-day accuracy and precision of the method for the determination of puerarin (*n*=5)

|  | Plasma (ng/mL) | | | Brain homogenate (ng/mL) | | |
| --- | --- | --- | --- | --- | --- | --- |
|  | 11260 | 1126 | 56.3 | 2252 | 281.5 | 28.15 |
| Accuracy (RE%) | | | | | | |
| Intra-day | 4.7 | 3.3 | 10.9 | 5.9 | 4.2 | 11.8 |
| Inter-day | 5.0 | 6.5 | 12.7 | 8.2 | 5.8 | 13.3 |
| Precision (RSD%) | | | | | | |
| Intra-day | 9.1 | 4.3 | 14.8 | 10.1 | 2.8 | 12.5 |
| Inter-day | 6.0 | 1.6 | 12.3 | 6.9 | 8.5 | 13.9 |
