# Supplementary material for: Enhancing both oral bioavailability and brain penetration of puerarin using borneol in combination with preparation technologies
Source: Drug Deliv. 2017 Feb 6;24(1):422–9. doi: 10.1080/10717544.2016.1259372 (PMC8241152; doi:10.1080/10717544.2016.1259372)
Supplement: Appendix_A._Supplementary_method_for_the_BBB.doc [file IDRD_A_1259372_SM7159.doc]

**Appendix A. Supplementary method for the BBB**

***Isolation and purification of primary cultures of BMECs***

Primary rat BMECs were prepared from the brain of 2-3 weeks old rats, as previously described. Briefly, meninges, large vessels and white matter were removed carefully and the grey matter was minced into small pieces of approximately 1.0 mm^3^ in ice-cold D-Hanks. After centrifuged at 150 (×g) for 3 min, the precipitation layer was added with trypsin（2.5 mg/mL）and digested at 37℃ for 5 min. Cold DMEM-F12（1:1）medium with 20% FBS was added to terminate the digestion and then it was centrifuged at 150（×g） for 5min. The precipitate was re-suspended in 25% BSA and centrifuged at 600（×g）for 15 min. The microvessels obtained were then digested in collagenase type II (1.0 mg/mL) and DNase (1.5 mg/mL) at 37 ℃ for 1 h and the medium with 20% FBS was added to suspend it. This new suspension was filtered through a 10-mm-pore-size nylon mesh and washed with medium containing 10% FBS. After the filtrate was centrifuged at 150 (×g) for 5min, the precipitate layer was re-suspended with a medium with 20% FBS, basic ﬁbroblast growth factor（bFGF, 1.0 ng/mL)，heparin（100.0 mg/mL）, penicillin (100.0 U/mL), L-glutamine (0.6mg/mL) and streptomycin (100.0 mg/mL). After the density adjustment, the microvessel endothelial cells were seeded on 25 cm^2^ plastic dishes pre-coated with gelatin (15.0 mg/mL) and incubated at 37 ℃ and 5% CO_2_. The culture medium was changed every 2 days. When the confluence reached 80% (the 6th day), the endothelial cells were further purified with trypsin (2.5 mg/mL) and EDTA (0.2 mg/mL) solution.

***Isolation and purification of primary cultures of rat brain astrocyte***

Rat cerebral astrocytes were obtained from brain cortices of 2 day old rats, as described in previous reports. Meninges, large vessels and white matter were removed carefully and grey matter pieces were dissociated mechanically into small pieces in ice-cold D-Hanks. The cortical pieces were disaggregated in trypsin (2.5 mg/mL) diluted with Ca^2+^/Mg^2+^ free PBS at 37℃ for 2 min. After centrifuged at 150(×g) for 5 min, the precipitate was re-suspended in the medium with 10% FBS. The suspension was fil-tered through a 10-mm-pore-size nylon mesh and washed by medium with 10% FBS. Finally, the filtrate was centrifuged at 150 (×g) for 5 min and then re-suspended with culture medium containing 10% FBS, penicillin (100.0 U/mL), streptomycin (100.0 mg/mL) and plated on 25 cm^2^ plastic dishes pre-coated with poly-L-lysine (0.1 mg/mL) at 37 ℃ and 5% CO_2_. On the second day, it was changed with new medium. Then the culture medium was changed every 2 days. When the conﬂuence reached 80% (the 4-5th day), the plastic dish was shaken at 220 rpm for 18h at 37 ℃to purify the astrocytes. The purified astrocytes were passaged by a brief treatment with trypsin (2.5 mg/mL)-EDTA (0.2 mg/mL) solution and the second passage was used to establish the in vivo NVU model.

***Construction of in vitro BBB models***

The *in vitro* BBB model was established by co-culturing primary rat BMECs and cerebral astrocytes on opposite side of Transwell membrane inserts. Primary rat BMECs were prepared from the brain of 2 - 3 weeks old rats and rat cerebral astrocytes were obtained from brain cortices of 2 day old rats, as described in previous reports (Xue et al., 2013). Before starting the co-culture, all cells were adapted to the same medium DMEM-F12 (1:1) containing 20% FBS, L-glutamine (0.6 mg/mL), penicillin (100.0 U/mL) and streptomycin (100 mg/mL). When the confluence gradually increased up to 90%, the two types of cells were used to establish the model. Astrocytes (1.5 × 10^5^ cells/cm^2^) were seeded into the matching well under the insert membrane. Then, the Transwell membrane was placed upside down in the incubator. Depending on the surface tension, the medium could not flow out and the astrocytes gradually adhered to the outer side of poly-L-lysine-coated (10.0 mg/mL) insert membrane. After 4 h, the insert was placed in the well, and BMECs (1.0 × 10^5^ cells/cm^2^) were seeded in the inner side of the insert membrane coated with gelatin (30.0 mg/mL). The day when the endothelial cells were plated was defined as day zero in vitro (day 0). All cells were cultured for 7 days with daily change of medium before analysis. As negative controls, BMECs and astrocytes were cultured on the inserts, respectively.

***Evaluation of the barrier integrity***

Transepithelial electrical resistance (TEER) and permeability studies with sodium fluorescein (SF) were used to check the integrity of the *in vitro* BBB model. TEER was measured using an EVOM instrument (Millipore Corporation, USA). TEER of coated, but cell-free filters was subtracted from the measured TEER values of the models shown as Ω × cm^2^. The flux of SF across the endothelial cells was determined as follows: briefly, the medium were removed from the apical and the basal compartments and replaced with 1mL and 2 mL DEME-F12 medium, respectively. After incubation for 30 min at 37 ℃, the DEME-F12 medium in apical compartments was replaced with 1 mL DMEM-F12 medium containing 2.162 μg/mL SF. At 15, 30, 60, 90 and 120 min, 100 μL medium was removed from the basal compartment and replaced with 100 μL of fresh DMEM-F12 medium. The concentrations of SF in samples were determined by ELx800 fluorescence microplate reader (Bio Tek, USA; excitation: 420 nm, emission: 545 nm). Transendothelial permeability coefficient (*P_e_*) was calculated according to the following equation: *P*_app_ = d*Q/*d*t* × 1/*AC*_0_.

Where d*Q/*d*t* is the transport rate (μg/s), *C*_0_ is the initial drug concentration on the apical side (μg/mL), and *A* is the surface area of the membrane filter (cm^2^).

Table 1. The TEER and *P*_app_ of SF for Astrocytes, BMECs and the BBB model（*n*=3）.

| Cell | TEER (Ω·cm^2^) | *P*_app_ (×10^-6^cm·s) |
| --- | --- | --- |
| Astrocytes monolayers | 274.1 ± 61.0 ^a^ | 14.56 ± 0.11 ^b^ |
| BMECs monolayers | 317.7 ± 35.6 ^a^ | 14.80 ± 0.21 ^b^ |
| The BBB model | 345.7 ± 37.9 | 7.25 ± 0.07 |

^a^*P* < 0.05 compared with the BBB model.

^b^*P* < 0.01 compared with the BBB model.

The TEER of co-culture model increased to 345.7 Ω·cm^2^ and was significantly higher (*P* < 0.05) than that of BMECs culture (317.7 Ω·cm^2^) or astrocytes (274.1 Ω·cm^2^) alone. The *P*_app_ of SF in the co-culture model (7.25 ×10^-6^ cm·s) was significantly lower (*P* < 0.01) than that in the BMECs (14.56 ×10^-6^ cm·s) and Astrocytes cultured (14.80 ×10^-6^ cm·s) alone. It was concluded that the *in vitro* BBB model with a good barrier function was established.
